# Impulsiveness does not prevent cooperation from emerging but reduces its occurrence: an experiment with zebra finches

**DOI:** 10.1038/s41598-017-09072-w

**Published:** 2017-08-17

**Authors:** Camille Chia, Frédérique Dubois

**Affiliations:** Département de Sciences Biologiques, Université de Montréal, Montréal, Qc Canada

## Abstract

Reciprocal altruism, the most probable mechanism for cooperation among unrelated individuals, can be modelled as a Prisoner’s Dilemma. This game predicts that cooperation should evolve whenever the players, who expect to interact repeatedly, make choices contingent to their partner’s behaviour. Experimental evidence, however, indicates that reciprocity is rare among animals. One reason for this would be that animals are very impulsive compared to humans. Several studies have reported that temporal discounting (that is, strong preferences for immediate benefits) has indeed a negative impact on the occurrence of cooperation. Yet, the role of impulsive action, another facet of impulsiveness, remains unexplored. Here, we conducted a laboratory experiment in which male and female zebra finches (*Taenyopigia guttata*) were paired assortatively with respect to their level of impulsive action and then played an alternating Prisoner’s Dilemma. As anticipated, we found that self-controlled pairs achieved high levels of cooperation by using a Generous Tit-for-Tat strategy, while impulsive birds that cooperated at a lower level, chose to cooperate with a fixed probability. If the inability of impulsive individuals to use reactive strategies are due to their reduced working memory capacity, thus our findings might contribute to explaining interspecific differences in cooperative behaviour.

## Introduction

The evolution and maintenance of cooperation among non-kin has posed a major challenge to evolutionary biologists and social scientists. Reciprocal altruism, one of the most probable explanations for cooperation among unrelated individuals, has been modelled as a Prisoner’s Dilemma^1^. In this two-player game, each player can choose to either defect or cooperate. Although mutual cooperation provides both players with higher payoffs than mutual defection, the highest payoff is achieved by defecting while the opponent cooperates. As a result, defection is the optimal strategy when the players interact only once. However, cooperation could emerge and be maintained when the game is repeated provided that the opponents, who expect to play together for an unknown number of interactions, adopt a conditional strategy like Tit-for-Tat (TFT)^2^. TFT, which consists of cooperating on the first round of the iteration and then doing whatever the opponent did on the previous round, is successful because it never defects first, retaliates when the opponent defects but forgives when the opponent reverts to cooperation^2^. On the other hand, TFT cannot correct mistakes, and for that reason performs poorly in a noisy environment, where other strategies like “win-stay, lose-shift” (“Pavlov”) or Generous TFT (also called “Firm but Fair”) are the most successful in the simultaneous and alternating game, respectively^3, 4^. Contrary to the standard game in which both players decide simultaneously to cooperate or defect, the alternating game considers that the players choose their move in turns, which reflects more accurately natural situations^5^.

While reciprocity is common in human societies, available data suggest that this mechanism would be very rare in animal societies^6–9^ probably because it requires, to evolve, specialized cognitive abilities that most species would not possess^9, 10^. In particular, several authors have pointed out that temporal discounting (i.e. strong preferences for immediate benefits), one aspect of impulsiveness, would prevent animals from achieving high levels of cooperation in an Iterated Prisoner’s Dilemma (IPD). Specifically, cooperation in an IPD is advantageous only on the long-term, but animals, because of their strong preference for immediate rewards, would systematically give in to the short-term temptation of defecting^11–14^. Several studies strongly support the idea that animals would be unable to sustain high levels of cooperation because they discount the value of delayed rewards^15–21^. For example, experimental studies with humans and rats have reported that individuals with higher levels of self-control in a temporal-discounting task (i.e. less impulsive individuals) were more likely to cooperate against an opponent that was adopting a TFT strategy^16, 20, 21^. Other studies have also demonstrated that both pigeons and blue jays were capable of maintaining high levels of cooperation but only when the effect of temporal discounting was reduced^17–19^ (e.g. when the birds could not access food rewards before having completed a series of trials). Finally, Baker and Rachlin^15^ showed that the level of cooperation displayed by pigeons was higher when the delay between two consecutive trials was short.

Previous studies that have investigated the link between impulsiveness and cooperation, however, have concentrated to date on the role of temporal discounting (or impulsive choice), whereas there is growing evidence that impulsiveness has different facets that would reflect different cognitive and neural processes^22, 23^. Notably, another facet of impulsiveness is impulsive action, which reflects the failure to inhibit an inappropriate response to prepotent stimuli^23, 24^. Though its role on cooperation remains unexplored, high levels of impulsive action could impede cooperation by affecting the capacity of individuals to react to their opponent’s past behaviour. Indeed, it has been argued that individuals that are unable to inhibit an automatic response, and hence have high levels of impulsive action, would be less capable of exhibiting behavioural flexibility^25^. Since high levels of cooperation can only be achieved by using conditional strategies, then cooperation should be less likely to evolve in species exhibiting high levels of impulsive action. The degree of impulsive action, which can be measured using the detour-reaching task^26, 27^, can vary widely both among species^24^ and among individuals of the same species^28^. In order to explore the effect of impulsive action on cooperation in an IPD, we then used impulsive-assorted pairs of zebra finches (*Taeniopygia guttata*) and tested whether their ability to achieve and sustain high levels of cooperation in an alternating Prisoner’s Dilemma was affected by their level of impulsive action. We conducted our experiment with established pair bonds, because it is necessary for cooperation to be maintained that individuals expect to interact repeatedly with each other. Accordingly, St-Pierre *et al*.^29^ have demonstrated that zebra finches were capable of maintaining high levels of cooperation in an IPD game, but only when they were interacting with their social partner, with whom they have established a long-lasting relationship.

## Results

The rate of cooperation (i.e. the proportion of cooperative choices) was significantly higher in self-controlled pairs (mean ± SEM: 0.41 ± 0.10) than in impulsive pairs (0.27 ± 011; LME: F_1,21_ = 7.880, *P* = 0.011). On the other hand, we detected no significant difference between self-controlled and impulsive pairs in their cumulated payoffs (self-controlled pairs: 83.57 ± 1.52 seeds/session, impulsive pairs: 78.40 ± 1.75 seeds/session; LME: F_1,21_ = 2.184 *P* = 0.154). Yet, at the individual level, the sex X impulsiveness interaction had a significant effect on the payoffs received (LME: F_1,55_ = 4.495, *P* = 0.039), revealing that the effect of sex depended on the level of impulsiveness of the birds. In impulsive pairs, the payoffs indeed differed considerably between males and females (males: 43.73 ± 1.87, females: 34.67 ± 1.55; F_1,19_ = 12.279, *P* = 0.002), while both pair members did not received significantly different payoffs in self-controlled pairs (males: 42.62 ± 1.43, females: 40.95 ± 1.69; F_1,28_ = 0.470, *P* = 0.499).

**Figure 1:**
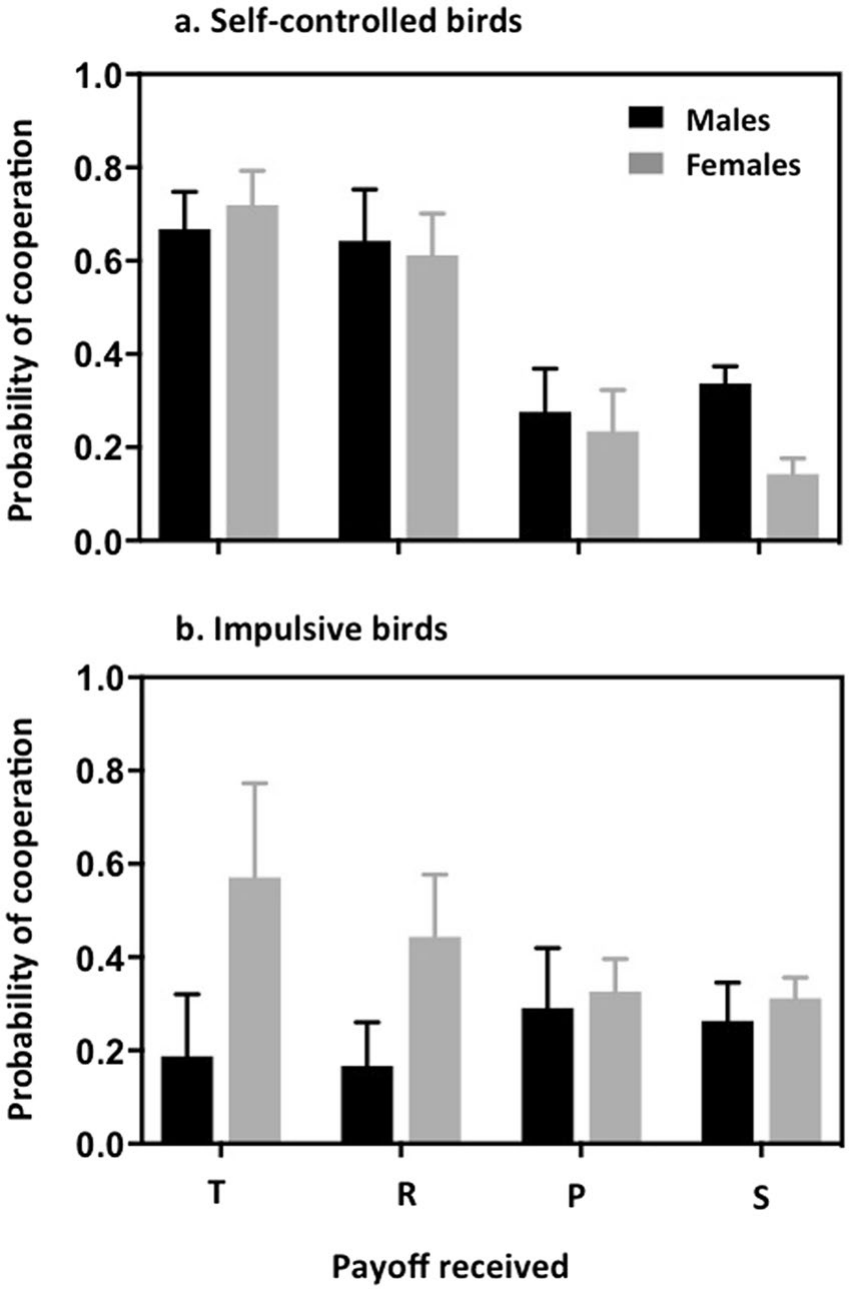
Mean (±SEM) probabilities of cooperation after the birds had received payoffs T, R, P and S, in self-controlled (panel a) and impulsive (panel b) birds. In both cases, the black and grey columns correspond to males and females, respectively.

Those differences were attributed to differences in the strategy used by self-controlled and impulsive males and females (Fig. 1). Specifically, we found that the probability of cooperating after receiving payoff R (i.e. after both partners had cooperated) was significantly higher in self-controlled pairs than in impulsive pairs. Similarly, self-controlled birds had a stronger tendency to cooperate after receiving payoff T (i.e. after the subject defected and the opponent cooperated) compared to impulsive individuals, but the effect of impulsiveness was only marginally significant (Tables 1 and 2). On the contrary, the probability that the birds continue cooperating after S (i.e. after the subject cooperated and the opponent defected) was not affected by their level of impulsiveness, while the probability that the birds switch from defection to cooperation after P depended on both their sex and impulsiveness, as revealed by a significant sex X impulsiveness interaction (Tables 1 and 2). Self-controlled males indeed cooperated more frequently than self-controlled females after both had defected (males: 0.34 ± 0.04, females: 0.14 ± 0.03; F_1,28_ = 15.387, *P* = 0.001) while the probability of cooperating after P did not differ between males and females in impulsive pairs (males: 0.26 ± 0.08, females: 0.31 ± 0.04; F_1,18_ = 2.057, *P* = 0.169).

**Table 1:** Observed and expected probability that a subject chooses to cooperate in a given trial after having received payoffs T, R, P and S, respectively.

	T	R	P	S
Self-controlled birds (mean ± SEM)	0.62 ± 0.15	0.71 ± 0.05	0.24 ± 0.03	0.25 ± 0.13
Impulsive birds (mean ± SEM)	0.28 ± 0.07	0.37 ± 0.08	0.29 ± 0.04	0.31 ± 0.09
TFT	1.0	1.0	0	0
Generous TFT	1.0	1.0	α	α
Pavlov	0	1.0	1.0	0

While a TFT player systematically defects after its opponent’s defection, an individual playing a Generous TFT strategy is expected to continue cooperating with a probability α after both payoffs P and S.

**Table 2:** Results from linear mixed effects analyses examining the effects of impulsiveness, sex, their interaction and testing day on the probability of cooperating after receiving payoffs T, R, P and S. Effects that remained significant after correction with the Benjamini–Hochberg procedure are shown in bold.

	df	F	*P*
*T*			
Impulsiveness	1,39	4.921	0.086
Sex	1,39	0.960	0.389
Impulsiveness × Sex	1,39	1.343	0.324
Day of testing	13,39	0.356	0.976
*R*			
**Impulsiveness**	**1,39**	**5.849**	**0.020**
Sex	1,39	2.597	0.115
Impulsiveness × Sex	1,39	1.872	0.179
Day of testing	13,39	0.671	0.768
*P*			
Impulsiveness	1,54	2.989	0.090
Sex	1,54	1.845	0.180
**Impulsiveness × Sex**	**1,54**	**6.971**	**0.011**
Day of testing	13,54	1.219	0.292
*S*			
Impulsiveness	1,39	1.640	0.208
Sex	1,39	0.013	0.909
Impulsiveness × Sex	1,39	0.027	0.870
Day of testing	13,39	1.247	0.285

## Discussion

Contrary to previous laboratory studies in which animals only succeeded in maintaining high levels of cooperation after they had been trained to cooperate under a mutualistic matrix^18, 19, 29, 30^, cooperation evolved in our experiment in all pairs. Thus, our findings indicate that alternating the roles of donor and recipient, instead of making simultaneous choices, makes cooperation more likely to evolve in animals. The inability of animals to implement complex strategies might explain their incapacity of establishing cooperation in the simultaneous Prisoner’s Dilemma. Indeed, strategies that respond both to their previous last move and that of their opponent (i.e. memory-2 strategies like Pavlov) or that required even longer memories have been shown to be more efficient in the simultaneous Prisoner’s Dilemma than memory-1 strategies that react only to their opponent’s last move^4, 31^. Experimental evidence, however, indicates that animals would adopt mainly simple strategies^18, 29^ that perform poorly in the simultaneous Prisoner’s Dilemma but allow cooperation to evolve in the alternating game. Our results also support this conclusion since we found that both impulsive and self-controlled birds used simple strategies that do not require important cognitive abilities. On the other hand, we detected significant differences between impulsive and self-controlled birds both in their propensity to cooperate and in their strategy.

Specifically, consistent with our expectation, cooperation occurred more frequently between self-controlled partners than between impulsive ones, despite the fact that mutual cooperation provided greater long-term benefits than mutual defection. Thus, differences in impulsive action and impulsive choice both contribute in explaining differences in cooperative behaviour. Moreover, we found that self-controlled individuals used a Generous TFT-like strategy: they had a high probability of cooperating (i.e. between 65% and 70%) after their partner’s cooperation that was independent of their own strategy (i.e. after playing R or T). In addition, contrary to TFT players, they were forgiving their partner 25% of the time after receiving payoffs P and S. As expected by theory, self-controlled birds, therefore, made choices contingent to their partner’s behaviour. Yet, we detected differences within self-controlled pairs between males and females in their probability of cooperating, with males being more inclined than females to cooperate after receiving payoffs P, T and S, though the difference was not significant for T and S. This finding is consistent with results from previous studies showing that females exhibit greater impulsive choices compared to males^32, 33^ and then underlines the importance of considering the sex of the subjects when studying cooperation.

By contrast, impulsive birds chose to cooperate with a fixed probability of around 30%, which was independent of their partner’s previous decision. The most probable explanation for this finding is that impulsive individuals would be incapable of using conditional strategies because they are relatively inflexible in their behaviour. Indeed, the ability to inhibit ineffective prepotent responses, which we used in our study to assess impulsiveness, is thought to be crucial to promoting behavioural flexibility^25^. Accordingly, Amici *et al*.^27^ reported that primates living in more cohesive groups had lower performances on inhibition tasks compared to species with a high degree of fission-fusion dynamics. Furthermore, although the differences were not significant, we noticed that impulsive females were more willing to cooperate than males, especially after having received payoffs R and T (i.e. after the male cooperated), which resulted in lower cumulated payoffs received by females compared to males. One potential reason that could explain why females had a higher probability than males of reciprocating (i.e. of cooperating after their partner had cooperated) is that they would have more to loose if their partner ends the sequence of play or switches to another partner.

Finally, although the birds differed among each other in their propensity to cooperate according to their level of impulsiveness, the rate of cooperation never exceeded 50%, even for self-controlled birds. Such relatively low levels of cooperation certainly resulted from the fact that the players, in our experiment, were facing a partner that was free to choose to either cooperate or defect on each round (and whose choices, therefore, were at least to some extent unpredictable), instead of playing against a stooge using a programmed strategy^18–20^. Yet, when individuals are uncertain about what their opponent will do on the next move, they have no incentive to cooperate and hence should defect^34^. Consistent with this prediction, a number of studies have reported that high levels of cooperation could be achieved only when the subjects played against an opponent that was adopting a fixed TFT strategy, while high levels of defection were attained when the opponent was using a pseudo-random strategy with a 50% probability of defecting^16, 20, 21^.

In conclusion, the present study suggests that high degrees of impulsive action might impede cooperation, though affecting the ability of individuals to flexibly adjust their behaviour to their partner’s decision. Yet, additional studies with larger sample sizes are needed to confirm these results and better understand the underlying mechanisms. In particular, we still know little about the neural mechanism that would make impulsive individuals unable to implement conditional strategies and it remains unclear to what extent our findings might explain why cooperation occurs so rarely in animal societies. We suggest that the observed differences between impulsive and self-controlled pairs in their propensity to cooperate would be related to differences in their working memory, which supposedly developed quite late in phylogenies and has been found to be related to impulsive action in rats^35^.

## Materiel and Methods

### Subjects

We used 28 adult zebra finches (11 males and 17 females) aged approximately four years: all individuals were used in the impulsiveness experiment, while we used only 8 of them (4 males and 4 females) in the cooperation experiment. All the birds came from a local breeder (Exotic Wings & Pet Things, St Clements, Ontario, Canada).

During the impulsiveness experiment, all the birds were housed in same-sex cages (38 × 38 × 48 cm) with a maximum of 4 individuals per cage, under a 12:12 h dark:light photoperiod and at a temperature of 23 ± 1 °C. Then following pair formation, each pair was housed in an individual cage (38 × 38 × 48 cm) under a 10:14 h dark:light photoperiod and at a temperature of 23 ± 1 °C.

Outside the experimental sessions, the birds had unlimited access to fresh water, seeds, cuttlefish bone, oyster shell and egg food supplement. All procedures were in compliance with the guidelines of the Canadian Council for Animal Care and were approved by the committee of ethics on animal use of the University of Montreal (animal care permit #16–040).

### Impulsiveness experiment

**Figure 2:**
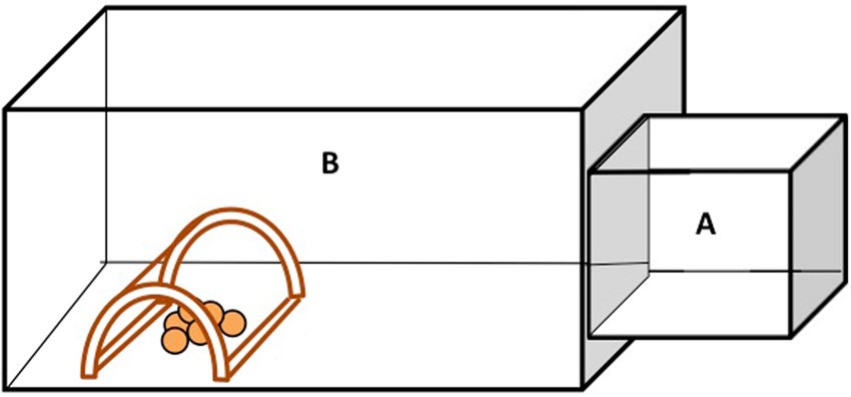
Side view of the experimental apparatus used to assess impulsiveness. The bird was first confined during 20 seconds in the observation chamber (**A**) then, it could enter the experimental chamber (**B**) where 5 millet seeds were placed inside either a transparent or an opaque cylinder.

We assessed the level of impulsiveness of each bird using the detour-reaching task. The birds were tested individually in an experimental apparatus that was composed of 2 chambers, an observation chamber (11 × 11 × 11 cm) and an experimental chamber (56 × 40 × 30 cm), which were separated by a transparent removable partition (Fig. 2).

For the training phase, the birds were first familiarized with finding 5 millet seeds hidden inside an opaque half-cylinder that was placed in the experimental chamber. The bird was introduced in the observational chamber and after 20 seconds the experimenter gently lifted the transparent partition so that it could have access to the experimental chamber. Each bird was trained to make a detour on the left or on the right to reach the food. The training was considered completed when the bird was able to reach the seeds directly by making a detour in less than 10 seconds following its entrance to the experimental chamber.

Then, the opaque cylinder was replaced by a transparent one and each bird experienced 10 consecutive trials. For each trial, we noted whether the bird had succeeded (i.e. performed a detour straight away to get access to the seeds) or failed (i.e. bumped into the cylinder). A bird that bumped into the cylinder, and, hence was unsuccessful at reaching the food, was allowed to continue searching for food until it performed the correct detour response. At the end of each trial (i.e. once the bird had consumed the 5 millet seeds), the experimenter gently removed the bird from the experimental chamber to put it back in the observation chamber where it was confined during 20 seconds before the next trial began. Once the birds had completed 10 trials, we could measure a score of impulsiveness depending on the percentage of failed trials. For all the birds tested, the level of impulsiveness varied between 0 and 80% with an average ( ± SEM) percent of failed trials that was equal to 30.71% ± 5.89 (Supplementary Fig. 1). For the cooperation experiment, the birds which had a low percentage of failures (i.e. between 0% and 30%) were then considered self-controlled, whereas those with a high percentage of failures (i.e. between 70% and 100%) were considered impulsive.

### Cooperation experiment

#### Pair formation

We used 8 birds (4 males and 4 females) that were selected among the 28 individuals we previously measured the level of impulsive action: half of the birds (2 males and 2 females) were impulsive whereas the other birds were self-controlled. The birds were matched to form assorted pairs, so that we had 2 self-controlled pairs and 2 impulsive pairs. Before being tested, we ensured that all the birds had established a strong relationship with their social partner by verifying that they displayed clumping or preening behaviours towards their mate. We started the training session 5 days after pair formation.

#### Experimental apparatus and procedure

**Figure 3:**
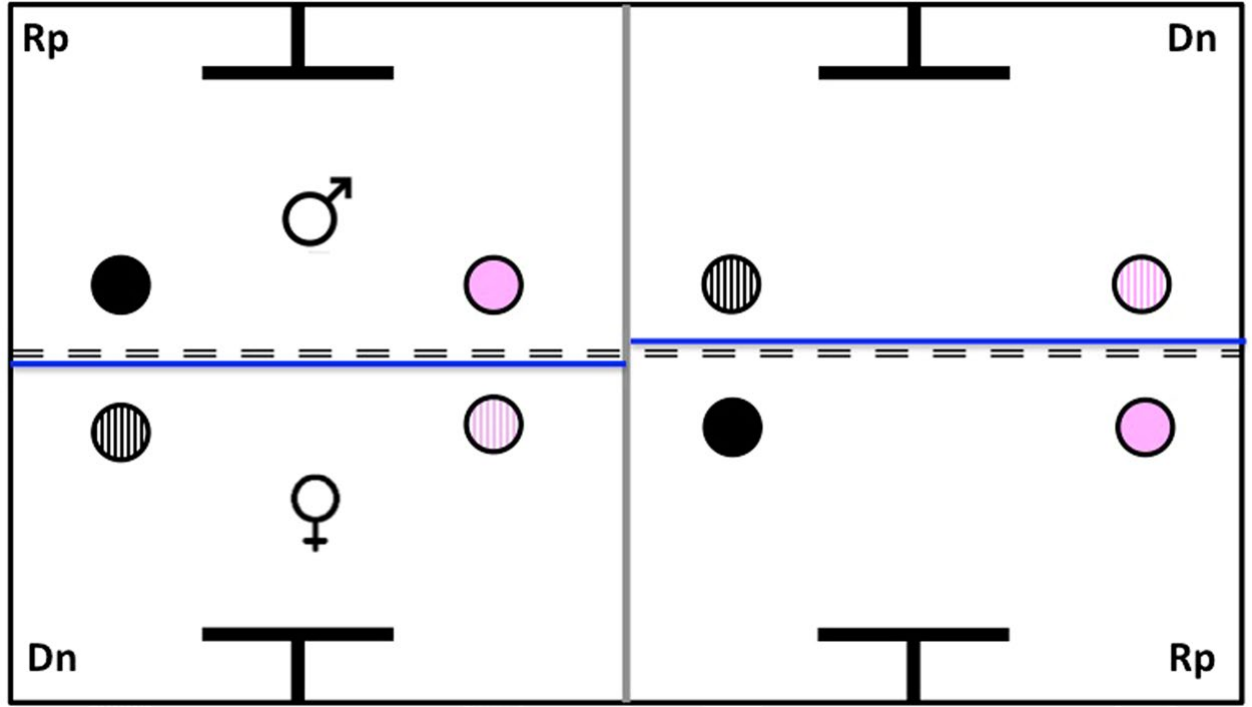
Top view of the experimental apparatus replicating an Alternating Prisoner’s Dilemma game. The two pair members - one in the donor chamber (Dn) and the other in the recipient chamber (Rp) - were separated only by a grid represented by a dotted line. In both chambers, the pink feeder represents the decision to cooperate while the black feeder represents the decision to defect. The crosshatched feeders placed in the Dn chamber were covered by a transparent lid, contrary to those placed in the Rp chamber. After the donor (the female in that case) has made a decision, the removable partition, which is represented by a grey line, was lifted, thereby allowing the birds to move to the adjacent chamber and switch roles.

The experimental apparatus (Fig. 3) replicated a two-player, two-choice game based on the alternating Prisoner’s Dilemma in which each bird could either cooperate or defect alternately. It was composed of 4 chambers (40 × 32 × 30 cm each), 2 donor chambers and 2 recipient chambers. During a given trial, one bird was assigned the role of the donor, while the other bird, which was housed in the opposite chamber facing the donor’s chamber, was assigned the role of the recipient. Since both partners were separated only by a grid, they could always see, hear and interact with each other. A T-shaped perch and 2 coloured feeders, each representing the decision to cooperate or defect, were placed in the chamber of both players. The 2 feeders of the same colour were positioned facing each other in the donor and recipient chambers. Yet, for the donor, the feeders were filled before it had to make a decision and were covered by a transparent lid. On the contrary, the recipient’s feeders were uncovered and empty. To stress the difference between the two types of chambers, we also added a piece of blue cardboard behind the feeders in the donors’ chambers.

Once the 2 birds had entered their respective chamber, we waited that the donor had made a decision by positioning itself in front of one of the 2 feeders for at least 3 seconds. Then, the experimenter first lifted the lid covering the feeder chosen by the donor and then provided the recipient with the corresponding number of seeds via a plastic tube that was put directly in the assorted feeder. Thirty seconds after the 2 birds had finished eating, the experimenter lifted the removable partition so that the birds could move into the adjacent chamber and thus switch roles.

#### Training and experimental treatments

Prior to testing, the birds were trained to move in the apparatus and to choose between the 2 feeders (i.e. a blue and a green feeder) when in the donor chamber. At the beginning of the training, both players received the same amount of seeds regardless of whether the donor chose the blue or the green feeder. Then, to insure that all the pairs could adjust their behaviour to maximize their immediate gain, they were exposed to a mutualism treatment, in which the feeder representing the decision to cooperate (i.e. the blue one) provided both players with 4 millet seeds, while the feeder representing the decision to defect (i.e. the green one) provided both players with only one seed. The position of the feeders was randomly chosen at the beginning of each testing day and then switched every 4 decisions. Each pair experienced 20 trials per day after 4 hours of food deprivation, and this series of trials was repeated until both birds chose to cooperate, at least 9 over 10 times.

Then, the 4 feeders were replaced by 2 pink and 2 black ones, and each pair was exposed to an alternating Prisoner’s Dilemma treatment. We used feeders of different colours for the 2 treatments to increase the probability that the birds rapidly learned that payoffs have changed when they experienced a new treatment. For the Prisoner’s Dilemma treatment, the feeder representing the decision to cooperate (i.e. the pink ones) provided the donor and the recipient with one and 5 seeds, respectively, while the feeder representing the decision to defect (i.e. the black ones) provided the donor and the recipient with 3 and zero seeds, respectively. Hence, when considering 2 consecutive decisions (i.e. one decision by each pair member alternately), the payoffs conform to a prisoner’s dilemma, with the payoff matrix given by:

$${\rm{PD}}=(\begin{array}{cc}R=6 & S=1\\ T=8 & P=3\end{array})$$

The parameter R denotes the number of seeds that each bird received when they both chose to cooperate one after the other, while P corresponds to their payoff when they were both defecting. Finally, when only one bird cooperated and the other defected, the rewards were S and T for the co-operator and the defector, respectively.

Each pair experienced 20 trials per day after 4 hours of food deprivation for 16 consecutive days. The first bird to make a decision was alternated between days, so that both the male and the female had an equal opportunity to decide first. During each trial we noted the birds’ decision as well as the number of seeds they received after every decision.

### Statistical analyses

For each pair, we calculated the rate of cooperation (i.e. the proportion of cooperative choices) as well as its cumulative gain during each testing day and then we tested whether both variables differed between impulsive and self-controlled pairs using linear mixed-effects (LME) models to account for repeated measurements within the same individuals. The rate of cooperation or the cumulative gain was entered as the dependent variable while impulsiveness and day of testing were entered as fixed factors and pair was included as a random factor.

To determine whether the strategy used by the birds was influenced by their sex or their level of impulsiveness, we also calculated for each bird the probability that it cooperated following T, R, P and S payoffs every testing day and then we ran a linear mixed-effects model; the probability of cooperation was entered as the dependent variable while the sex of the bird, its level of impulsiveness and the day of testing were considered as fixed factors and the bird was included as a random factor. The same procedure was used to test for individual difference in payoffs received. To control for multiple tests, we used the false discovery rate (FDR) method^36^. Contrary to the very conservative family-wise error rate (FWER) methods that control the chance of making even a single false rejection, the FDR method controls the fraction of wrong rejections among the rejected hypotheses, thus giving a better balance between type I and type II errors. This approach, therefore, is an appropriate solution for ecological studies in which repeated tests are performed^37–39^.

For each pair, we excluded from the analyses the days in which at least one pair member had a side bias (i.e. was automatically going towards the same side of the apparatus for all the trials regardless of the position of the feeders). Statistical analyses were performed with SPSS 23.0.

## Electronic supplementary material

Supplementary Figure 1
