# Supplementary material for: Impulsiveness does not prevent cooperation from emerging but reduces its occurrence: an experiment with zebra finches
Source: Sci Rep. 2017 Aug 17;7:8544. doi: 10.1038/s41598-017-09072-w (PMC5561265; doi:10.1038/s41598-017-09072-w)

IMPULSIVENESS DOES NOT PREVENT COOPERATION FROM EMERGING BUT REDUCES ITS OCCURRENCE: AN EXPERIMENT WITH ZEBRA FINCHES

Camille Chia & Frédérique Dubois

Supplementary Figure 1. Distribution of impulsiveness scores. Birds with a low percent of failures (i.e. equal or lower than 30%) were considered self-controlled, while those with a high percent of failures (i.e. equal or greater than 70 %) were considered impulsive.

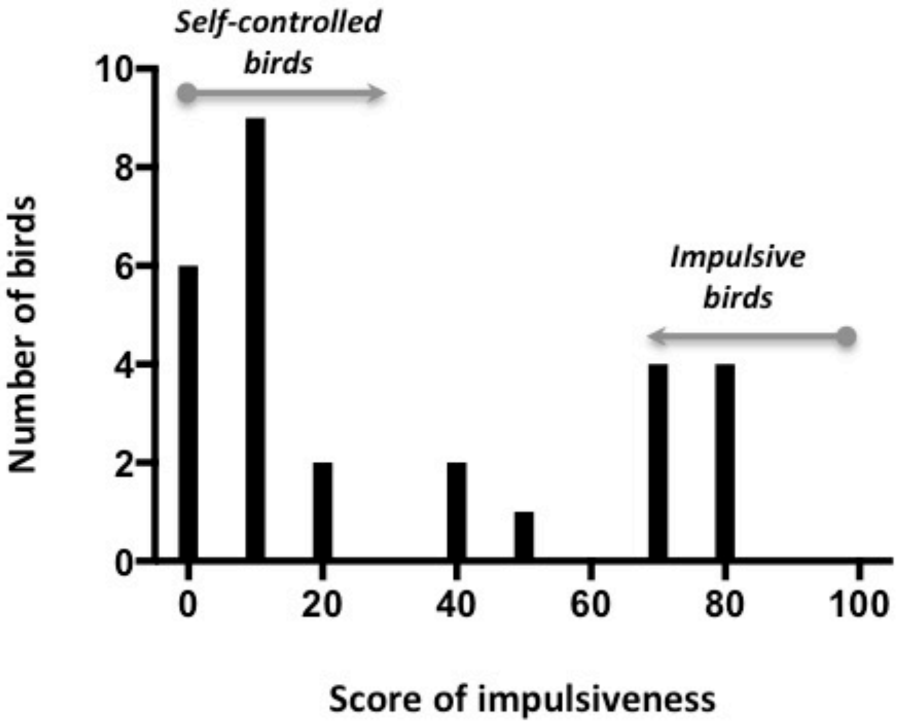

Supplement: Supplementary file 1 — Supplementary Figure 1 [file 41598_2017_9072_MOESM1_ESM.pdf]
